# Enhanced multimodal medical image fusion based on Pythagorean fuzzy set: an innovative approach

**DOI:** 10.1038/s41598-023-43873-6

**Published:** 2023-10-04

**Authors:** Maruturi Haribabu, Velmathi Guruviah

**Affiliations:** School of Electronics Engineering, Vellore Institute of Technology, Chennai, India

**Keywords:** Image processing, Computational biology and bioinformatics, Diseases, Medical research, Mathematics and computing

## Abstract

Medical image fusion is the process of combining a multi-modality image into a single output image for superior information and a better visual appearance without any vagueness or uncertainties. It is suitable for better diagnosis. Pythagorean fuzzy set (PFS)-based medical image fusion was proposed in this manuscript. In the first phase, a two-scale gaussian filter was used to decompose the source images into base and detail layers. In the second phase, a spatial frequency (SF)-based fusion rule was employed for detail layers to preserve the more edge-oriented details. However, the base layer images were converted into pythagorean fuzzy images (PFIs) using the optimum value obtained by pythagorean fuzzy entropy (PFE). The blackness and whiteness count fusion rule were performed for image blocks decomposed from two PFIs in the third phase. Finally, the enhanced fused image was obtained by reconstructions of fused PFI blocks, which performed the defuzzification process. The proposed method was evaluated on different datasets for disease diagnosis and achieved better mean (M), standard deviation (SD), average gradient (AG), SF, modified spatial frequency (MSF), mutual information (MI), and fusion symmetry (FS) values than compared to state-of-art methods. This advancement is important in the field of healthcare and medical imaging, including enhanced diagnostics and treatment planning.

## Introduction

In the medical field, many imaging technologies are widely used for disease detection, namely, MRI (magnetic resonance imaging), CT (computed tomography), MRA (magnetic resonance angiography), PET (positron emission tomography), SPECT (single photon emission computed tomography), weighted MR-T1, and MR-T2 images. These imaging technologies are easy to operate, inexpensive, and able to produce an enormous amount of information about a patient's health. Although these advantages, elucidating them still presents a significant difficulty. It can be challenging for even experienced radiologists to identify subtle nodules or differentiate between similar lesions. Additionally, manual disease screening is labour-intensive and time-consuming. In recent years, with the rapid growth of disease screening technology and management, it has been important to develop an effective computer-aided diagnosis (CAD) system that shortens the diagnosis time. Early diagnosis is essential for rapid recovery and improved survival. Recent advances in computer-aided diagnosis (CAD) have shown significant potential for automatically identifying and detecting diseases in medical images. CAD is widely used to assist doctors in the efficient diagnosis of disease, treatment planning, and making decisions more accurately. In general, different modalities of medical imaging are widely used for the purpose of disease diagnosis in clinical applications^1^.

Usually, different categories of information, like tissue, bone, or organ functionality, are reflected from various modalities of medical images. CT images are non-invasive and able to clearly show and depict dense organ information like bones and fractions, but they are unable to capture the fine features of soft tissues. MRI scans are non-invasive and used for the examination of delicate tissue, but do not reveal the hard structures like bones. The weighted MR-T1 images reveal the fact, while the MR-T2 images provide water. Both PET and SPECT images are invasive, may provide functional data about organs, and offer information on blood flow at certain locations^1,2^. However, it can be concluded that single medical imaging is not appropriate in all aspects. Therefore, multimodal approaches merge medical images from different modalities to generate a new fused image, which can help doctors to make better judgments of patient’s conditions. For example, MRI and CT^3^ images provide soft and hard tissues. MR-T1 and MRA^4^ images show delicate tissues with lesion location. MRI and PET^5^ images provide information about soft tissue and functionality. MRI and SPECT^6^ images show soft tissue and cerebral blood flow information. Several image fusion algorithms have emerged that occur at the pixel level, feature level, and decision level. Among them, the independent data points are extracted from the modalities by feature-level fusion and then form two-point sets that can be adapted for connection. Decision-level fusion associates divergent inputs with a common phenomenon using decision labels. Furthermore, pixel-level fusion is divided into spatial and spectral domains^7–9^. The maximum, median, and minimum methods are part of the spatial domain methods.

These methods produce a degraded fusion image with unwanted noise. The statistical techniques are subspace methods, namely, principal component analysis (PCA)^10^ and independent component analysis (ICA)^11^, which are also included in the spatial approach, produces spectral distortions and low contrast. Pyramid-based decomposition methods^12–14^, such as the Gaussian pyramid and Laplacian pyramid, obtained fused images with loss of spatial data.

To address these drawbacks, wavelet transform-based fusion methods are introduced such as discrete wavelet transform (DWT)^15^, stationary wavelet transform (SWT)^16^, and duel-tree complex wavelet transform (DTCxWT)^17^. In addition, multiscale transforms such as curvelet and contourlet transforms were produce edge information in a fused image. These methods are not shift-invariant and have limited directionality. To overcome this, nonsubsampled contourlet transform (NSCT)^18^ and nonsubsampled shearlet transform (NSST)^19^ methods were employed.

Medical images are poor illuminated, as various structures are not visible and some parts are vague in nature. In image processing applications, the fuzzy set plays a tremendous role to improving the image quality in various aspects such as contrast, highlighting the region of interest, clear edges, etc. Zadeh proposed a mathematical tool known as fuzzy set in 1965^20^. Manchanda et al.^21^ proposed image fusion using fuzzy transform, which produces a fused image with edge distortions. Intuitionistic fuzzy set (IFS) is the generalized version of the fuzzy set, which was introduced by Atanassov in the year 1986^22^. IFS is used to remove vagueness and uncertainties by utilizing a hesitation degree.

Balasubramaniam et al.^23^, Tirupal et al.^24^ proposed intuitionistic fuzzy sets-related fusion methods, and these are used to remove vagueness but do not cover complete uncertainties. According to the aforementioned discussion, the major issues with the previous techniques contained undesirable artifacts, distortions, and unwanted errors. Therefore, this paper proposed a Gaussian filtering-based image fusion method that leads to a two-layer decomposition. The decomposition technique extracts significant features from the original images. As a result, the fused image contains more reliable information without distortions and artifacts, which aids clinical diagnosis. The base layer images were fused by using pythagorean fuzzy set (PFS) for high contrast with maximum information, and detailed layer images were fused based on spatial frequency for clarity representation. Lastly, the enhanced fused image can be reconstructed by summing the base and detailed layer fused images.

The major contribution of the proposed work is as follows:

- The two-layer decomposition model decomposes the original images into two layers: base and detail layers, for the extraction of structural and detailed information.
- To deal with uncertainty, a new pythagorean fuzzy approach is used for medical image fusion, which will produce an enhanced fused image without artifacts and uncertainties.
- The proposed fusion method produces better fusion results in terms of visual appearance and quantitative phenomena compared to other state-of-the art methods.

The construction of IFS includes both membership $\left( \mu \right)$, and non-membership $\left( \nu \right)$, with a hesitation margin $\left( \pi \right)$. Such that, $\mu + \nu \le 1$ and $\mu + \nu + \pi = 1$. The concept of IFSs offers a flexible framework for elaborating on vagueness and uncertainties. The IFS concept appears to be useful in many real time circumstances such as selection processes, medical diagnoses, multi-criteria decision-making, etc^25^. Where the situations $\mu + \nu \ge 1$ occur in IFSs, which lead to construct a new mathematical tool, called Pythagorean fuzzy set (PFS)^26^. This was a new method to deal with vagueness and uncertainties more accurately and sufficiently than the IFSs by considering membership $\left( \mu \right)$, and non-membership $\left( \nu \right)$ grades, with satisfying conditions $$\mu + \nu \le 1\begin{array}{*{20}c} {\left( {or} \right)\mu + \nu \ge 1} \\ \end{array}$$, and also it follows that $\mu^{2} + \nu^{2} + \pi^{2} = 1$, where $\pi$ is the PFS index or indeterminacy degree. The Pythagorean fuzzy set was attracted by many researchers in various application areas such as segmentation, enhancement, medical diagnosis, decision- making, etc.

The remaining paper is arranged as follows: Section “Pythagorean fuzzy approach for image fusion” includes the pythagorean fuzzy approach for image fusion. Section “Image enhancement” describes a proposed medical image fusion in grayscale and color. Section “Experimental analysis” presents the experimental analysis. In Section “Fusion results and analysis”, fusion result are analyzed. Lastly, the conclusion part is presented in Section “Conclusion”.

## Pythagorean fuzzy approach for image fusion

### Construction of a Pythagorean fuzzy set

Let $H$ be a finite set. A Pythagorean fuzzy set (PFS)^27^ $G$ in $H$, is defining as:

1 $$G = \left\{ {h,\mu_{G} \left( h \right),\nu_{G} \left( h \right)\left| {h \in H} \right.} \right\}\,{\text{or}}\,G = \left\{ {\left\langle {\frac{{\mu_{G} \left( h \right),\nu_{G} \left( h \right)}}{h}} \right\rangle \left| {h \in H} \right.} \right\}$$

Where $\mu_{G} \left( h \right),\nu_{G} \left( h \right):h \to [0,1][0,1]$ are the membership and non-membership degrees of the element $h \in H$ of PFSs, with the condition that $0 \le \left( {\mu_{G} \left( h \right)} \right)^{2} + \left( {\nu_{G} \left( h \right)} \right)^{2} \le 1$, and the indeterminacy degree $\pi_{G} \left( h \right)$ of PFS reflects the uncertainty of membership and non-membership functions, which is defined as:

2 $$\pi_{G} \left( h \right) = \sqrt {1 - \left[ {\left( {\mu_{G} \left( h \right)} \right)^{2} + \left( {\nu_{G} \left( h \right)} \right)^{2} } \right]}$$

The concept of PFSs and IFSs are illustrated as in Table 1:

**Table 1:** IFSs and PFSs^27^.

IFS	PFS
$\mu_{G} \left( h \right) + \nu_{G} \left( h \right) \le 1$ $0 \le \left( {\mu_{G} \left( h \right)} \right) + \left( {\nu_{G} \left( h \right)} \right) \le 1$ $\pi_{G} \left( h \right) = 1 - \left[ {\left( {\mu_{G} \left( h \right)} \right) + \left( {\nu_{G} \left( h \right)} \right)} \right]$ $\left( {\mu_{G} \left( h \right)} \right) + \left( {\nu_{G} \left( h \right)} \right) + \left( {\pi_{G} \left( h \right)} \right) \le 1$	$\mu_{G} \left( h \right) + \nu_{G} \left( h \right) \le 1$ or $\mu_{G} \left( h \right) + \nu_{G} \left( h \right) \ge 1$ $0 \le \left( {\mu_{G} \left( h \right)} \right)^{2} + \left( {\nu_{G} \left( h \right)} \right)^{2} \le 1$ $\pi_{G} \left( h \right) = \sqrt {1 - \left[ {\left( {\mu_{G} \left( h \right)} \right)^{2} + \left( {\nu_{G} \left( h \right)} \right)^{2} } \right]}$ $\left( {\mu_{G} \left( h \right)} \right)^{2} + \left( {\nu_{G} \left( h \right)} \right)^{2} + \left( {\pi_{G} \left( h \right)} \right)^{2} \le 1$

### Pythagorean fuzzy image

PFS is an extension version of the IFS^30^. The construction of PFS is described as:

3 $$N\left( {\mu_{G} \left( h \right)} \right) = m^{ - 1} \left( {m\left( 1 \right) - m\left( {\mu_{G} \left( h \right)} \right)} \right)$$

Let us consider an increasing function^31^ as:

$$m\left( {\mu_{G} \left( h \right)} \right) = \frac{{\mu_{G} \left( h \right)}}{{\alpha + \left( {1 - \alpha } \right)\mu_{G} \left( h \right)}}$$, with $$m\left( 0 \right) = \frac{0}{{\alpha + \left( {1 - \alpha } \right) \times 0}} = 0$$, and $$m\left( 1 \right) = \frac{1}{{\alpha + \left( {1 - \alpha } \right) \times 1}} = 1$$.

With $m^{ - 1} \left( {\mu_{G} \left( h \right)} \right) = \frac{{\mu_{G} \left( h \right)\alpha }}{{1 - \mu_{G} \left( h \right)\left( {1 - \alpha } \right)}}$ (using inverse function).

So, $$N\left( {\mu_{G} \left( h \right)} \right) = m^{ - 1} \left( {\frac{1}{{\alpha + \left( {1 - \alpha } \right)}} - \frac{{\mu_{G} \left( h \right)}}{{\alpha + \left( {1 - \alpha } \right)\mu_{G} \left( h \right)}}} \right)$$.

After solving, we obtain

4 $$N\left( {\mu_{G} \left( h \right)} \right) = \Psi \left( {\mu_{G} \left( h \right)} \right) = \frac{{\alpha^{2} \left[ {1 - \mu_{G} \left( h \right)} \right]}}{{\alpha^{2} \left[ {1 - \mu_{G} \left( h \right)} \right] + \left( {\mu_{G} \left( h \right)} \right)}},\alpha > 0$$

Where $$N\left( 1 \right) = 0,N\left( 0 \right) = 1.$$

Based on the IFS, the membership degree of PFS can be estimated as:

5 $$\mu_{G}^{PFS} \left( h \right) = 1 - \frac{{\alpha^{2} \left[ {1 - \mu_{G} \left( h \right)} \right]}}{{\alpha^{2} \left[ {1 - \mu_{G} \left( h \right)} \right] + \left( {\mu_{G} \left( h \right)} \right)}}$$

The estimation of non-membership degree of PFS is as:

6 $$\nu_{G}^{PFS} \left( h \right) = \frac{{\alpha^{2} \left[ {1 - \mu_{G}^{PFS} \left( h \right)} \right]}}{{\alpha^{2} \left[ {1 - \mu_{G}^{PFS} \left( h \right)} \right] + \left( {\mu_{G}^{PFS} \left( h \right)} \right)}}$$

Finally, the indeterminacy degree of PFS estimated as:

7 $$\pi_{G}^{PFS} \left( h \right) = \sqrt {1 - \left[ {\left( {\mu_{G}^{PFS} \left( h \right)} \right)^{2} + \left( {\nu_{G}^{PFS} \left( h \right)} \right)^{2} } \right]}$$

As mentioned above, α is not a fixed value for all the images and was optimized by pythagorean fuzzy entropy (PFE). In this article, pythagorean fuzzy entropy was suggested by Peng X^32^ and its mathematical formulated as follows:

8 $$PFE\left( {G;\alpha } \right) = \frac{1}{{\left| {A \times B} \right|}}\sum\limits_{a = 0}^{A} {\sum\limits_{b = 0}^{B} {\frac{{\left( {\pi_{G}^{PFS} \left( {h;\alpha } \right)} \right)^{2} + 1 - \left| {\left( {\mu_{G}^{PFS} \left( {h;\alpha } \right)} \right)^{2} - \left( {\nu_{G}^{PFS} \left( {h;\alpha } \right)} \right)^{2} } \right|}}{{\left( {\pi_{G}^{PFS} \left( {h;\alpha } \right)} \right)^{2} + 1 + \left| {\left( {\mu_{G}^{PFS} \left( {h;\alpha } \right)} \right)^{2} - \left( {\nu_{G}^{PFS} \left( {h;\alpha } \right)} \right)^{2} } \right|}}} }$$

PFE is calculated by using Eq. (8) for $\alpha$ values ranging from [0.1–1.0]. Similarly, the highest value of PFE correspond to the $\alpha$ value, is treated as the optimum value as shown in Table 2, and is denoted by:

9 $$\alpha_{opt} = \mathop {\max }\limits_{\alpha } \left( {PFE\left( {G;\alpha } \right)} \right)$$

**Table 2:** Pythagorean fuzzy entropy (PFE) values and α values.

Database	Pair	Source images	PFE values	Alpha (α) Range [0.1–1.0]										α_opt_ value
				0.1	0.2	0.3	0.4	0.5	0.6	0.7	0.8	0.9	1	
1	1	a1	Pythagorean fuzzy entropy (PFE) values	0.0678	0.1059	0.1457	0.1867	0.2230	0.2548	0.2880	**0.3104**	0.2986	0.2636	0.8
		a2		0.0530	0.0861	0.1219	0.1613	0.2003	0.2358	0.2682	0.2978	**0.3170**	0.3150	0.9
	2	a3		0.0453	0.0701	0.0996	0.1342	0.1689	0.2009	0.2298	0.2574	0.2809	**0.2845**	1.0
		a4		0.0589	0.0938	0.1336	0.1745	0.2100	0.2400	0.2693	**0.2897**	0.2773	0.2443	0.8
	3	a5		0.0787	0.1137	0.1500	0.1900	0.2279	0.2603	0.2855	0.3031	**0.3082**	0.2914	0.9
		a6		0.1091	0.1531	0.2000	0.2442	0.2791	0.3071	**0.3255**	0.3223	0.2996	0.2648	0.7
	4	a7		0.0602	0.0995	0.1455	0.1924	0.2311	0.2612	0.2853	0.3026	**0.3058**	0.2764	0.9
		a8		0.1022	0.1621	0.2077	**0.2396**	0.2276	0.1986	0.1697	0.1444	0.1231	0.1037	0.4
2	1	a1		0.1904	0.4401	0.6114	**0.6737**	0.6517	0.5868	0.5073	0.4251	0.3498	0.2836	0.4
		a2		0.1269	**0.1463**	0.1289	0.1034	0.0843	0.0712	0.0616	0.0545	0.0492	0.0448	0.2
	2	a3		0.1843	0.2591	0.3187	0.3509	**0.3603**	0.3435	0.3065	0.2567	0.2054	0.1610	0.5
		a4		0.0605	0.1080	0.1623	0.2124	0.2553	0.2890	0.3130	**0.3166**	0.2930	0.2587	0.8
3	1	a1		0.2124	0.2687	0.3158	0.3338	0.3381	0.3443	0.3578	0.3757	**0.3870**	0.3730	0.9
		a2		0.3686	0.4909	0.5467	**0.5476**	0.5437	0.5241	0.4270	0.3281	0.2442	0.1806	0.4
4	1	a1		0.0416	0.0589	0.0793	0.1035	0.1265	0.1464	0.1629	**0.1712**	0.1652	0.1435	0.8
		a2		0.0734	0.1314	0.1773	**0.1846**	0.1766	0.1668	0.1567	0.0989	0.0626	0.0334	0.4
	2	a3		0.0502	0.0895	0.1321	0.1740	0.2101	0.2388	0.2640	**0.2813**	0.2733	0.2475	0.8
		a4		0.0353	0.0431	0.0501	0.0552	0.0604	0.0673	0.0753	0.0849	0.0905	**0.0913**	1.0
	3	a5		0.0399	0.0602	0.0916	0.1231	0.1514	0.1766	0.1951	0.2037	**0.2044**	0.1994	0.9
		a6		0.0269	0.0362	0.0420	0.0429	0.0428	0.0438	0.0460	0.0486	0.0517	**0.0551**	1.0
	4	a7		0.0649	0.1084	0.1628	0.2148	0.2597	0.2966	0.3233	0.3393	**0.3463**	0.3450	0.9
		a8		0.0543	0.0745	0.0871	0.0914	0.0946	0.1008	0.1093	0.1180	0.1239	**0.1280**	1.0
5	1	a1		0.1950	0.2400	0.2970	0.3312	0.3500	0.3620	**0.3723**	0.3719	0.3456	0.3031	0.7
		a2		0.0617	0.1221	0.1906	0.2462	0.2855	**0.3168**	0.3103	0.2608	0.2116	0.1682	0.6
	2	a3		0.0737	0.1104	0.1588	0.2038	0.2397	0.2687	0.2959	**0.3012**	0.2775	0.2463	0.8
		a4		0.0646	0.1323	0.2080	0.2705	0.3148	**0.3456**	0.3432	0.3032	0.2568	0.2140	0.6
	3	a5		0.1558	0.1802	0.2413	0.2951	0.3349	0.3636	0.3881	**0.4071**	0.3907	0.3436	0.8
		a6		0.0517	0.1015	0.1633	0.2198	0.2639	0.2972	0.3242	**0.3397**	0.3234	0.2942	0.8
	4	a7		0.0730	0.1021	0.1463	0.1896	0.2275	0.2560	0.2793	0.3015	0.3038	**0.3193**	1.0
		a8		0.0314	0.0617	0.1032	0.1445	0.1804	0.1967	0.2130	0.2131	0.2277	**0.2358**	1.0
	5	a9		0.0653	0.0913	0.1309	0.1742	0.2122	0.2453	0.2791	**0.3136**	0.3135	0.2882	0.8
		a10		0.0463	0.0594	**0.0655**	0.0591	0.0457	0.0338	0.0252	0.0192	0.0151	0.0123	0.3

Significant values are in bold.

Substituting the $\alpha_{opt}$ value in Eq. (5), an pythagorean fuzzy image (PFI) is created.

## Image enhancement

The parameter, ($\lambda$)^33^ is a fuzzy hedge, and it varies according to the image. The $\lambda$ is used to modify the pythagorean fuzzy image $\mu_{G}^{PFS} \left( h \right)$ and also controls the contrast of the image, as in Eq. (10).

10 $$\mu_{new}^{PFS} \left( h \right) = \left( {\mu_{G}^{PFS} \left( h \right)} \right)^{\lambda } ,\begin{array}{*{20}c} {where} & { \in \left[ {1,\infty } \right]} \\ \end{array}$$

Then, the contrast stretching is applied on modified PFI using INT operator^30^, and is mathematically represented as:

11 $$\mu^{enh} \left( h \right) = \left\{ {\begin{array}{*{20}c} {2\left[ {\mu_{new}^{PFS} \left( h \right)} \right]^{2} } & {\begin{array}{*{20}c} ; & {\begin{array}{*{20}c} {if} & {0 \le } \\ \end{array} } \\ \end{array} \mu_{new}^{PFS} \left( h \right) \le 0.5} \\ {1 - 2\left[ {1 - \mu_{new}^{PFS} \left( h \right)} \right]^{2} } & {\begin{array}{*{20}c} ; & {\begin{array}{*{20}c} {if} & {0.5 \le } \\ \end{array} } \\ \end{array} \mu_{new}^{PFS} \left( h \right) \le 1} \\ \end{array} } \right.$$

The above-mentioned Eq. (11) forms the contrast enhanced image.

## Proposed fusion method description

**Figure 1:**
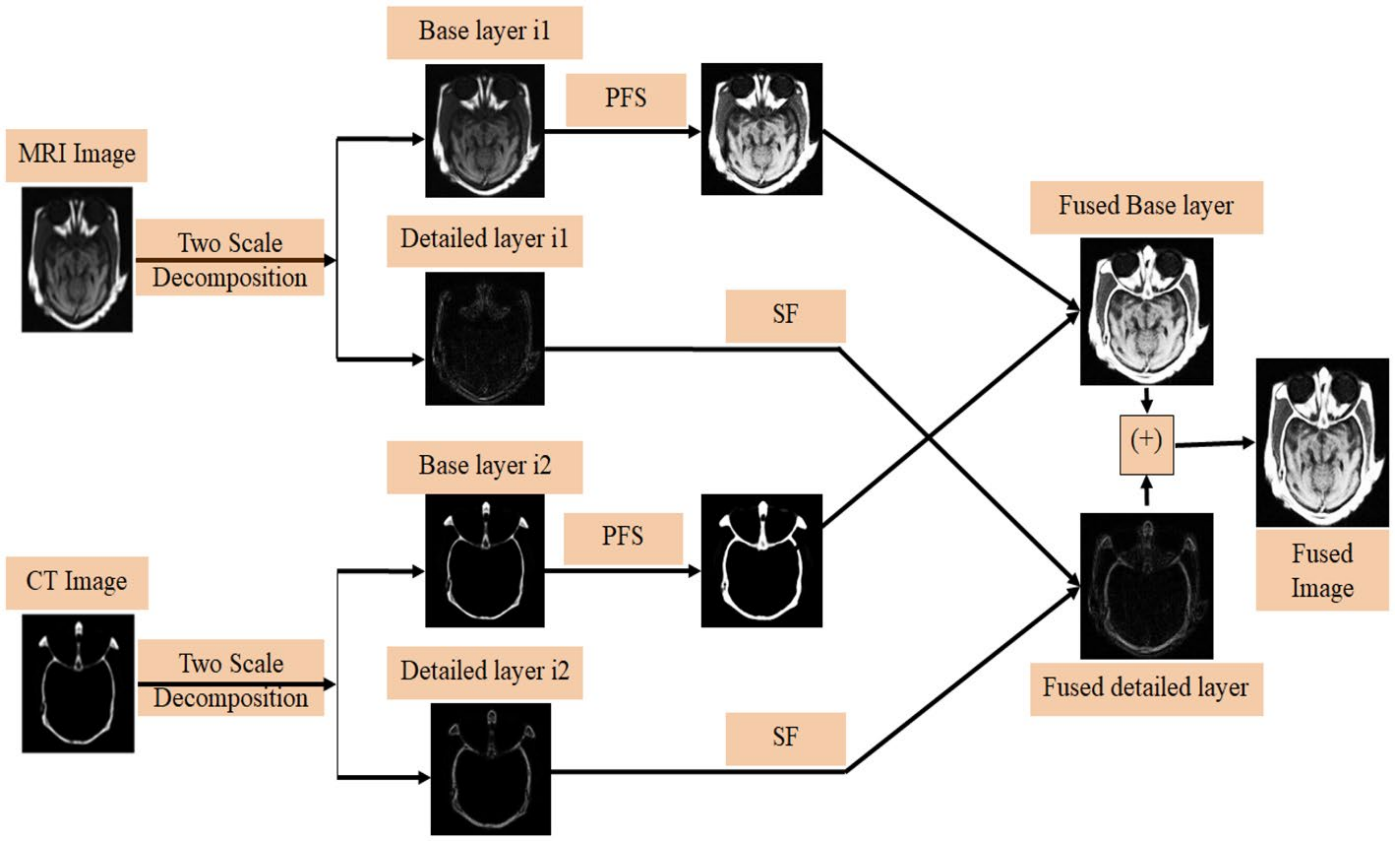
Schematic flowchart of the proposed grayscale image fusion.

This section describes the proposed fusion algorithm based on the pythagorean fuzzy set in detailed. Firstly, the pre-registered source images are decomposed into two layers by using a gaussian filter. Secondly, the base layers are fused based on PFSs, and the detailed layers are fused by using spatial frequency (SF). Lastly, the enhanced fused image is rebuilt by combining both the fused base and detailed layered images. The schematic flowchart of the proposed grayscale image fusion method is described in Fig. 1.

### Two-Layer decomposition using Gaussian filter

Let $X_{1}$ and $X_{2}$ are the two pre-registered input images of $A \times B$ dimension. Those images are decomposed into base and detail layers. The base layer is obtained by using (12), and the detail layer is obtained by removing the relevant base layer from the original images, as specified in (13).

12 $$\begin{array}{*{20}c} {Y_{i} = X_{i} \times G_{f} ,} & {i - 1,2} \\ \end{array}$$

13 $$\begin{array}{*{20}c} {Z_{i} = X_{i} - Y_{i} ,} & {i - 1,2} \\ \end{array}$$

### Base layer image fusion

**Figure 2:**
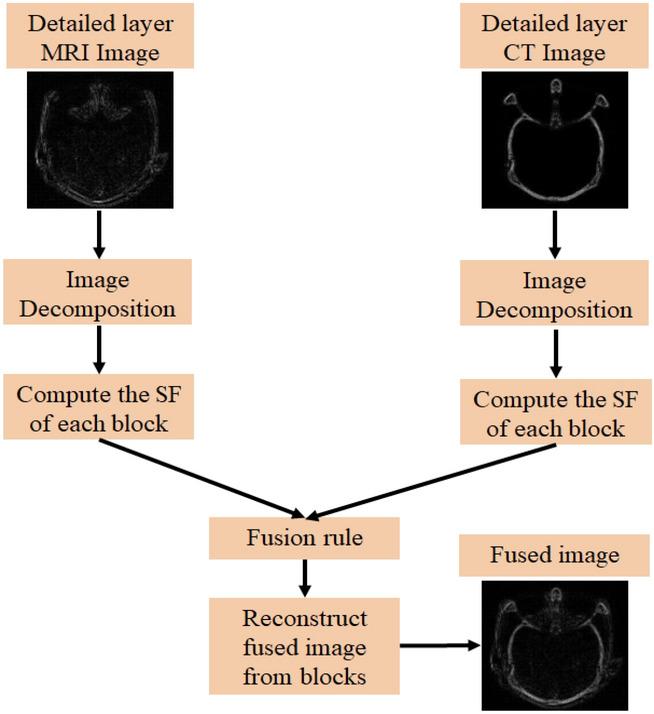
Schematic flowchart of the base layer fusion.

The base layer contains more structural information from the source images. In general, medical images are poor illuminated and some parts are not visible. As a result, PFS is used to enhance the fusion results and remove uncertainties. The schematic flowchart of the base layer fusion method is shown in Fig. 2. The detailed explanation of the base layer fusion algorithm is summarized as:

1. Initially, the base layer images are fuzzified separately using the membership function (14) with $A \times B$ dimension.
  14 $$\mu \left( {Y\left( {a,b} \right)} \right) = \frac{{Y_{ab} - Y_{\min } }}{{Y_{\max } - Y_{\min } }}$$
  where $Y\left( {a,b} \right)$ is the gray level value of the image at pixel $(a,b)$.$Y_{\max }$ and $Y_{\min }$ are the maximum and minimum gray level values of the image, respectively.
2. Calculate optimum value,$\alpha$, using Eqs. (8), (9) for two base layer images separately, and this $\alpha$ value varies from image to image.
3. Based on the optimum value,$\alpha$, the calculation of membership, non-membership, and indeterminacy degrees of two base layers images separately using Eqs. (5), (6), (7).
4. Finally, the enhanced PFI images are obtained from Eq. (11) and represented as $Y_{PFI1}$, and $Y_{PFI2}$.
5. Decompose the two PFI images $Y_{PFI1}$ and $Y_{PFI2}$ into $i \times j$, and* l*^*th*^ block of each image can be represented as $Y_{PFI1}^{l}$, and $Y_{PFI2}^{l}$.
6. The blackness and whiteness count of each block of the two images $Y_{PFI1}^{l}$ and $Y_{PFI2}^{l}$ are calculated by using min, max, and average operations, as given below:
  15 $$Y_{fused} \left( {i,j} \right) = \left\{ {\begin{array}{*{20}c} {\min \left( {Y_{PFI}^{l} (i,j),Y_{PFI}^{l} (i,j)} \right)} & {if} & {Blachness > Whiteness} \\ {\max \left( {Y_{PFI}^{l} (i,j),Y_{PFI}^{l} (i,j)} \right)} & {if} & {Blachness < Whiteness} \\ {\frac{{Y_{PFI}^{l} (i,j) + Y_{PFI}^{l} (i,j)}}{2}} & {if} & {otherwise} \\ \end{array} } \right.$$
7. The base layer fused image is obtained by reconstructing the $Y_{fused}$ image blocks and then perform defuzzification process to obtain a crisp image, which is the inversion of (14).
  16 $$Y_{B} = \left( {\left( {Y_{\max } - Y_{\min } } \right)*Y_{fused} + Y_{\min } } \right)$$

### Detail layer image fusion

**Figure 3:**
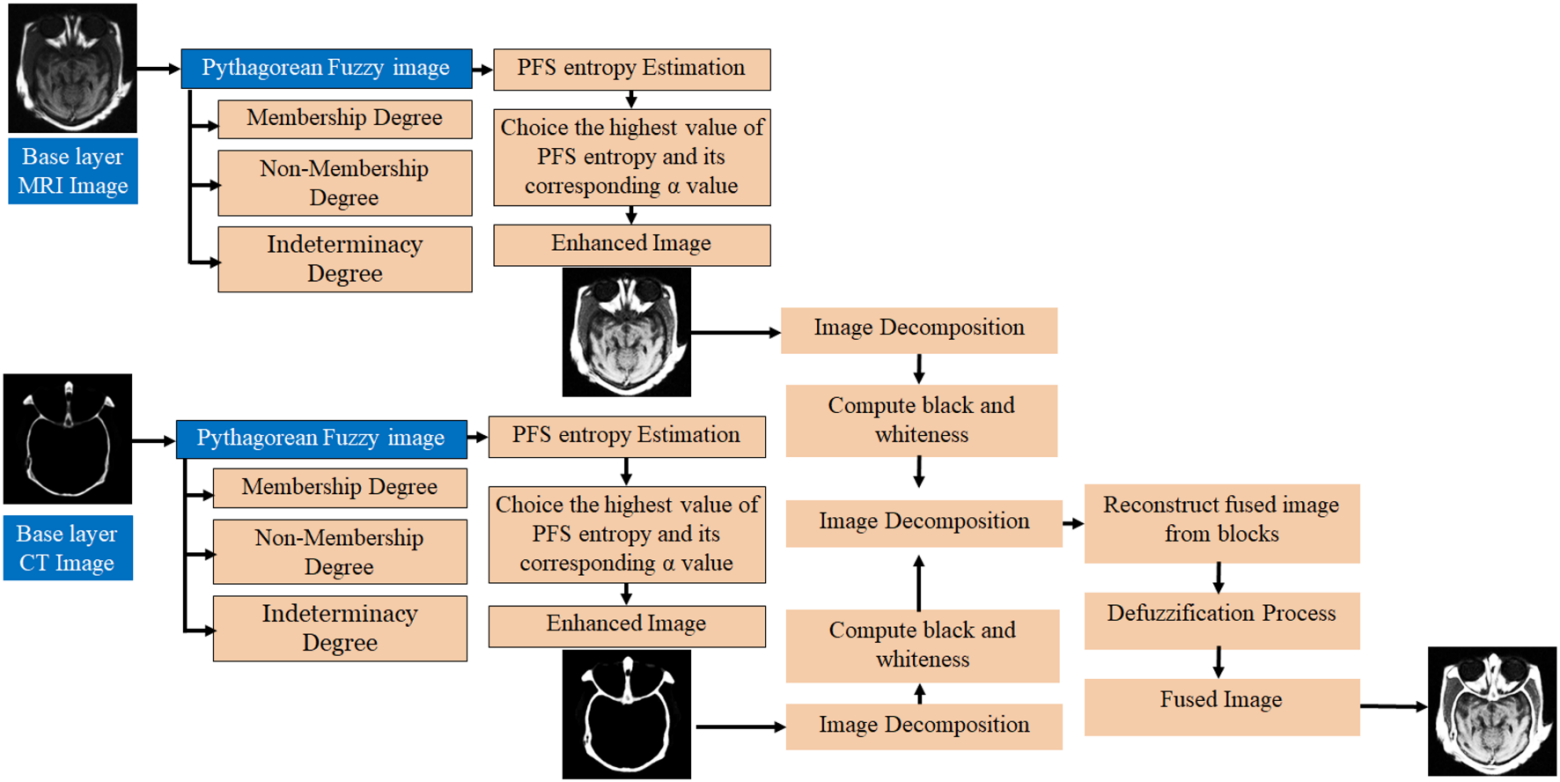
Schematic flowchart of the detailed layer fusion.

Detail layers contain information related to the edges of sub-images. In fact, spatial frequency (SF)^34^ measures the clarity and active levels of the image and is also susceptible to changes in image intensity. Due to this reason, the SF-based fusion rule is used to combine detail layers to obtain more edge information in the fused image. The schematic flowchart of the detail layer fusion method is shown in Fig. 3. The summary steps of the detail fusion algorithm are arranged as follows:

1. Decompose the detail layer sub images $Z_{i}$ into $i \times j$, and* l*^*th*^ block of each decomposed sub images are represented as $Z_{1k}$, and $Z_{2k}$.
2. Calculate the SF of each *lth* block and is given below:
  $$SF = \sqrt {\mathop {(RF)}\nolimits^{2} + \mathop {(CF)}\nolimits^{2} }$$
  $$RF = \sqrt {\frac{1}{i \times j}\sum\limits_{q = 1}^{i} {\sum\limits_{r = 2}^{j} {\mathop {\left[ {Z(q,r) - Z(q,r - 1)} \right]}\nolimits^{2} } } }$$
  $$CF = \sqrt {\frac{1}{i \times j}\sum\limits_{q = 2}^{i} {\sum\limits_{r = 1}^{j} {\mathop {\left[ {Z(q,r) - Z(q - 1,r)} \right]}\nolimits^{2} } } }$$
  where RF and CF are the row and column frequencies respectively.
3. These blocks are fused by using SF based fusion rule, and is given below:
  17 $$Z_{fk} = \left\{ {\begin{array}{*{20}c} {Z_{1k} \left( {q,r} \right)} \\ {Z_{2k} \left( {q,r} \right)} \\ {\left[ {\frac{{Z_{1k} \left( {q,r} \right) + Z_{2k} \left( {q,r} \right)}}{2}} \right]} \\ \end{array} \begin{array}{*{20}c} {SF\left( {Z_{ik} } \right) > SF\left( {Z_{2k} } \right) + TH} \\ {SF\left( {Z_{ik} } \right) < SF\left( {Z_{2k} } \right) + TH} \\ {others} \\ \end{array} } \right.$$
4. After the reconstruction of blocks, to obtain detailed fused image $Z_{D} .$

### Detail layer image fusion

Lastly, the final enhanced fused image $U$ is obtained by summing of both fused base ($Y_{B}$) and detailed ($Z_{D}$) images.

18 $$U = Y_{B} + Z_{D}.$$

### Color medical image fusion

**Figure 4:**
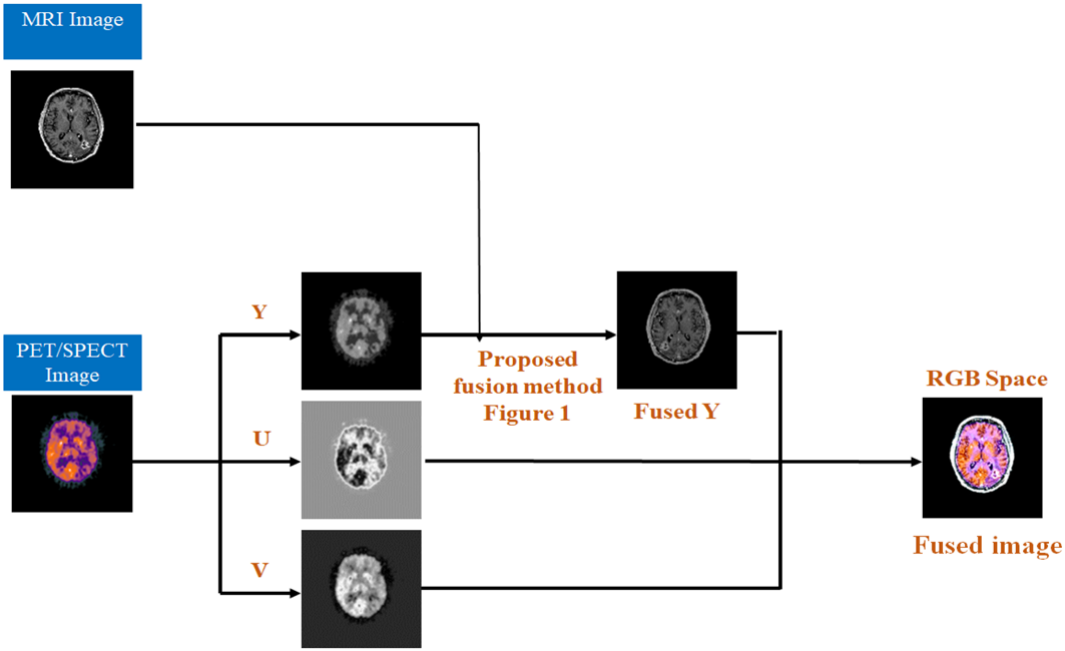
Schematic flowchart of the color medical image fusion.

In addition to grayscale image fusion, color images (PET and SPECT) were fused with MRI images, which play an essential role in clinical diagnosis and medical treatment. During the fusion process, PET and SPECT images are treated as RGB images. In the fusion of MRI images with PET/SPECT images, the most common method is to first divide the PET/SPECT image into R, G, and B channels and then combine them with the MRI image. However, this approach provides color distortions and makes the fusion process more complicated. Therefore, PET/SPECT images were converted into YUV color space, which is a highly efficient method for getting more complementary information used for better diagnosis. In this article, the color medical image fusion is described as follows: Firstly, the color images are transformed into YUV color space, such as luminance component (Y) and chrominance components (U and V). Secondly, through the proposed grayscale fusion method, perform the fusion process of MRI with the Y component to obtain a fused Y component, as shown in Fig. 4. Lastly, the fused Y and unchanged components (U and V) were transformed into RGB to obtain the enhanced fused color image.

## Experimental analysis

**Figure 5:**
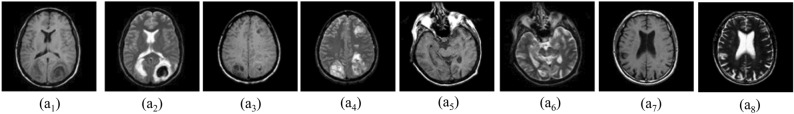
Database-1 Source Images^28^: (a_1_, a_2_) Pair-1, (a_3_, a_4_) Pair-2, (a_5_, a_6_) Pair-3, (a_7_, a_8_) Pair-4.

**Figure 6:**
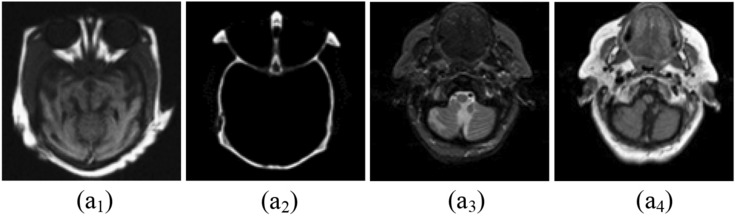
Database-2 Source Images^28,29^: (a_1_, a_2_) Pair-1, (a_3_, a_4_) Pair-2.

**Figure 7:**
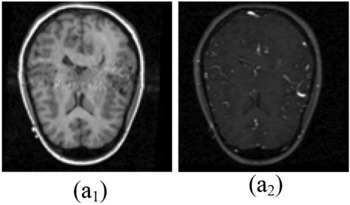
Database-3 Source Images^29^: (a_1_, a_2_) Pair-1.

**Figure 8:**
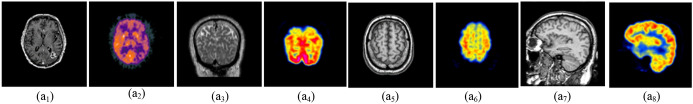
Database-4 Source Images^28^: (a_1_, a_2_) Pair-1, (a_3_, a_4_) Pair-2, (a_5_, a_6_) Pair-3, (a_7_, a_8_) Pair-4.

**Figure 9:**
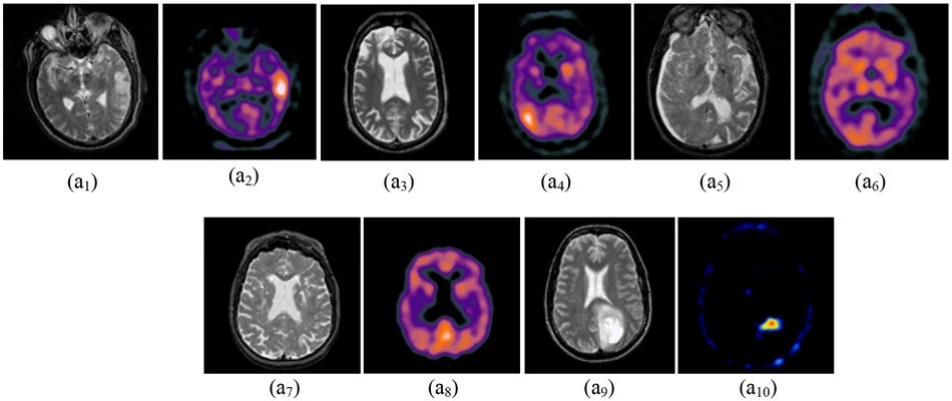
Database-5 Source Images^28^: (a1, a2) Pair-1, (a3, a4) Pair-2, (a5, a6) Pair-3, (a7, a8) Pair-4, (a9, a10) Pair-5.

**Figure 10:**
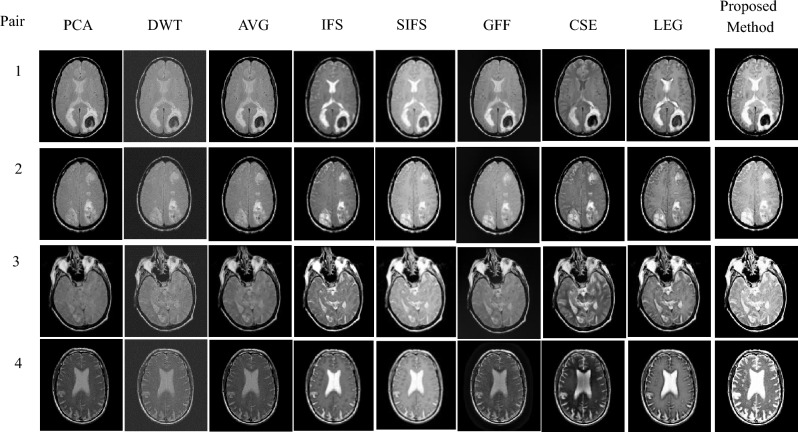
Fusion results of proposed and various existing fusion methods for Database-1.

**Figure 11:**
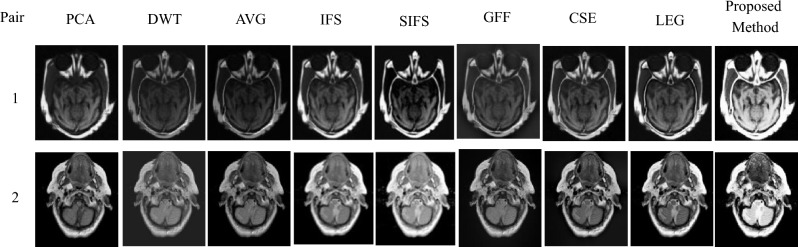
Fusion results of proposed and various existing fusion methods for Database-2.

**Figure 12:**
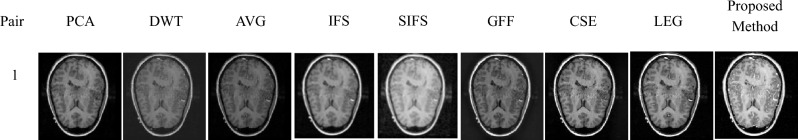
Fusion results of proposed and various existing fusion methods for Database-3.

**Figure 13:**
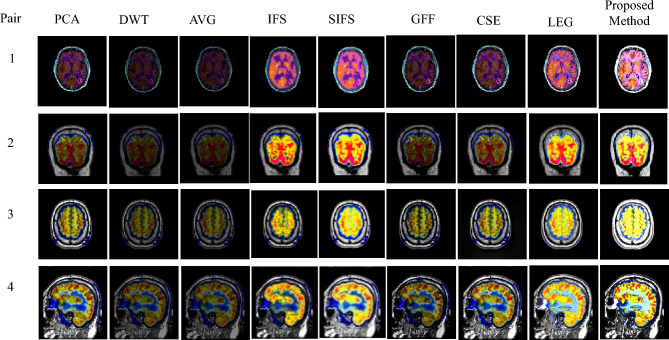
Fusion results of proposed and various existing fusion methods for Database-4.

**Figure 14:**
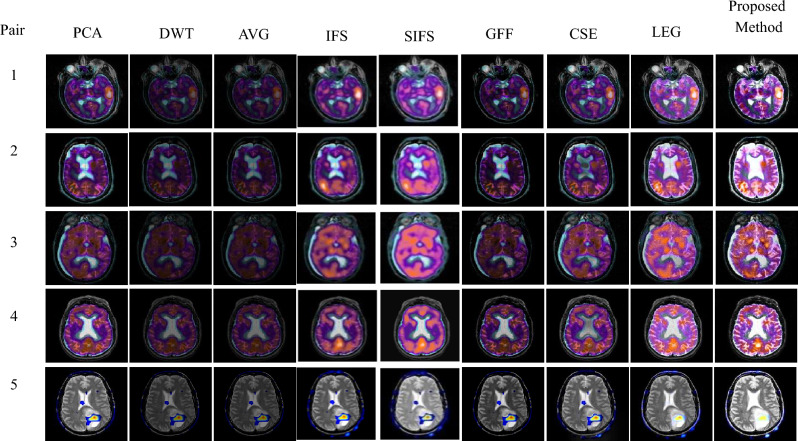
Fusion results of proposed and various existing fusion methods for Database-5.

The experimental results are executed on a personal PC with an i3 processor using MATLAB R2016a. The proposed method was evaluated on 16 medical image datasets, as shown in Figs. 5, 6, 7, 8, 9, which are publicly available^28,29^. These are obtained from different medical image modalities, namely, T1-MR and T2-MR, MRI and CT, T1-weighted MR and MRA, MRI and PET, and MR-T2 and SPECT images. The experimental analysis is carried out to confirm the efficiency and superiority of the proposed method. Furthermore, we have compared the proposed method's results with existing methods, and Figs. 10, 11, 12, 13 and 14 show how the proposed method effectively identifies the tumour regions.

### Objective measures

Objective metrics are used to assess the quality of the fused image. In this article, seven objective metrics were used, namely, mean (M)^34^, standard deviation (SD)^34^, average gradient (AG)^35^, spatial frequency (SF)^34^, modified spatial frequency (MSF)^36^, mutual information (MI)^37^, and fusion symmetry (FS)^38^. The higher value of each quality metric shows the efficiency of the fused image. The quality measures are listed as follows:

1. Mean (M) value represents the average pixel intensities, which shows the brightness of a fused image. It is denoted as,
  19 $$Mean\left( \mu \right) = \frac{1}{A \times B}\sum\limits_{a = 1}^{A} {\sum\limits_{b = 1}^{B} {\left[ {U_{ab} } \right]} }$$
  where $U_{ab}$ signifies the intensity values of a fused output image at $(a,b){th}$ pixel.
2. Standard deviation (SD) represents the degree of variation of gray level values in the fused image. The higher value of SD indicates no artifacts in the fused image and shows overall contrast. It is written as,
  20 $$SD = \sqrt {\frac{1}{A \times B}\sum\limits_{a = 1}^{A} {\sum\limits_{b = 1}^{B} {\left[ {U_{ab} - \mu } \right]^{2} } } }$$
3. Average gradient (AG) measures the directional changes of the pixels in the fused image, and is the formula as stated,
  21 $$AG = \frac{1}{{\left( {A - 1} \right)\left( {B - 1} \right)}}\sum\limits_{a = 1}^{A - 1} {\sum\limits_{b = 1}^{B - 1} {\sqrt {\frac{{\left[ {U(a,b) - U(a + 1,b)} \right]^{2} + \left[ {U(a,b) - U(a,b + 1)} \right]^{2} }}{2}} } }$$
4. Spatial frequency (SF) is the metric used to measure the quality and clarity of the fused image. The value of SF should be higher for better performance. The calculated formula is,
  22 $$SF = \sqrt {\left( {RF_{U} } \right)^{2} + \left( {CF_{U} } \right)^{2} }$$
  where
  $$RF_{U} = \sqrt {\frac{1}{A \times B}\sum\limits_{a = 1}^{A} {\sum\limits_{b = 2}^{B} {\left[ {U\left( {a,b} \right) - U\left( {a,b - 1} \right)} \right]^{2} } } }$$
  $$CF_{U} = \sqrt {\frac{1}{A \times B}\sum\limits_{a = 2}^{A} {\sum\limits_{b = 1}^{B} {\left[ {U\left( {a,b} \right) - U\left( {a - 1,b} \right)} \right]^{2} } } }$$
5. Modified spatial frequency (MSF) indicates the overall active levels of an image. It is calculated as
  23 $$MSF = \sqrt {\left( {RF_{U} } \right)^{2} + \left( {CF_{U} } \right)^{2} + \left( {DF{}_{U}} \right)^{2} }$$
  $$RF_{{U_{ab} }} = \sqrt {\frac{1}{{A \times \left( {B - 1} \right)}}\sum\limits_{a = 1}^{A} {\sum\limits_{b = 2}^{B} {\left[ {U\left( {a,b} \right) - U\left( {a,b - 1} \right)} \right]^{2} } } },$$
  $$CF_{{U_{ab} }} = \sqrt {\frac{1}{{\left( {A - 1} \right) \times B}}\sum\limits_{a = 2}^{A} {\sum\limits_{b = 1}^{B} {\left[ {U\left( {a,b} \right) - U\left( {a - 1,b} \right)} \right]^{2} } } }.$$
  and $$DF_{{U_{ab} }} = A_{{U_{ab} }} + B_{{U_{ab} }}$$.
  Where
  $$A_{{U_{ab} }} = \sqrt {\frac{1}{{\left( {A - 1} \right) \times \left( {B - 1} \right)}}\sum\limits_{a = 2}^{A} {\sum\limits_{b = 2}^{B} {\left[ {U\left( {a,b} \right) - U\left( {a - 1,b - 1} \right)} \right]^{2} } } }$$
  $$B_{{U_{ab} }} = \sqrt {\frac{1}{{\left( {A - 1} \right) \times \left( {B - 1} \right)}}\sum\limits_{a = 2}^{A} {\sum\limits_{b = 2}^{B} {\left[ {U\left( {a - 1,b} \right) - U\left( {a,b - 1} \right)} \right]^{2} } } }$$
6. Mutual information (MI) is the degree of dependency between two variables and indicates the amount of information is transferred between source images to fused image. It is denoted as,
  24 $$MI_{T} = MI_{{UX_{1} }} + MI_{{UX_{2} }}$$
  $$MI_{{UX_{1} }} = \sum\limits_{a = 1}^{A} {\sum\limits_{b = 1}^{B} {P_{{UX_{1} }} (a,b)\log \left( {\frac{{P_{{UX_{1} }} (a,b)}}{{P_{U} (a,b)P_{{X_{1} }} (a,b)}}} \right)} }$$
  $$MI_{{UX_{2} }} = \sum\limits_{a = 1}^{A} {\sum\limits_{b = 1}^{B} {P_{{UX_{2} }} (a,b)\log \left( {\frac{{P_{{UX_{2} }} (a,b)}}{{P_{U} (a,b)P_{{X_{2} }} (a,b)}}} \right)} }$$
  where $MI_{{UX_{1} }}$ is the mutual information between source image $X_{1}$ and fused image $U$, and $MI_{{UX_{2} }}$ is the mutual information between source image $X_{2}$ and fused image $U$ respectively.
7. Fusion symmetry (FS) is representing the symmetry of the fused image with respect to the source image. It is formulated as
  25 $$FS = 2 - \left| {\left( {\frac{{MI_{{UX_{1} }} }}{MI} - 0.5} \right)} \right|$$

The FS value is near to 2, which represents both source images equally contribute to producing a fused image. So, the fused image has better quality.

## Fusion results and analysis

Various existing methods are included in this experiment such as PCA, DWT, Average (AVG) method, IFS^23^, SIFS^24^, GFF^39^, CSE^40^, and JBF-LGE^41^, In this paper, all comparative methods are utilized and meet the criterion and terms according to their publications. The proposed method shows superiority and efficiency in all aspects.

### Visual assessment

Visual assessment is the subjective evaluation of a fused image that is based on human perception. Database-1 incorporates the fusion of T1-MR and T2-MR images. The combination of these two MR images produces adequate information and also highlights the fat and water content of the body. The experimental results of database-1 are shown in Fig. 10. According to the fusion results, the PCA method does not preserve white matter information. DWT and AVG-based resultant images are hazy and lack complementing information. Visually, the IFS-based method could not retain the key features of the source images. Also, SIFS fusion outputs lose a significant amount of energy from source images. Meanwhile, a GFF-based fused image has good edge information but fails to highlight the minute details. CSE and JBF-LEG-based methods produce a fused image with misplacement of primary details and distortions that may be observed. Finally, the proposed fusion method is superior to other methods and has greater luminance.

Database-2 contains MRI and CT image fusion. CT scans are made up of a sequence of X-ray images collected from various angles that show hard tissue, such as bone structure.

On the other hand, an MRI scan employs magnetic fields and radio waves to display the details of inside organs and delicate tissues. As a result, the combination of these two images produces a single fused image with more complementary information and salient features. The proposed and other existing results are clearly observed in Fig. 11. As a result of database-2, the PCA method does not produce CT details. The DWT and AVG methods are not good in terms of contrast. It should be emphasized that IFS generates undesired artifacts that cause distortion of local characteristics. SIFS causes the incorrect production of soft tissues. The GFF method produces undesirable artifacts and is missing complementary information. Some distortion is visible in the resultant fusion results of the CSE and JBE-LGE methods. However, the proposed fusion method outperforms other state-of-the-art methods in terms of contrast.

Database-3 comprises the fusion of T1-weighted MR and MRA images, with some diseases shown as a white structure observed in Fig. 12. The T1-weighted MR image provides delicate tissue data, but it fails to identify the abnormalities in the image. MRA images can detect abnormalities easily, but not soft tissue information.

Therefore, the combination of these two T1-MR and MRA images provides more reliable information in the integrated image, which can aid in medical diagnosis. First, the outcome of PCA shows the loss of white structure information. DWT and AVG provide a blurred and degraded fusion image. Moreover, the textural changes are not produced by the IFS and SIFS-based fusion results. Also, the soft tissue information is not visible in the GFF-based fused image. In addition, the anatomical information and structural details are misaligned for CSE and JBF-LEG fusion results. However, the proposed fusion method is superior compared to other methods in terms of appearance and clarity.

Database-4 addresses the MRI and PET fusion images as shown in Fig. 13. In this paper, the MRI image is perfectly registered with the corresponding PET image. The MRI image shows anatomical brain tissue information but no functional information, whereas the PET image shows the functionality of the brain but has a limited spatial resolution. Fusing these two images obtained more functional information without distortions. The proposed fused images have more quantitative complementary information showing the size of the tumor and are more visible than other existing methods, which can help doctors make better disease diagnoses at an earlier stage, as observed in Fig. 13. The resultant fused images of PCA have low anatomical information, while DWT and AVG methods produce distorted fused images with low contrast.

It is noted that the IFS and SIFS methods produced a fused image with color distortions. Sufficient information is not present in GFF-based fusion results. In addition, CSE results displayed the structural details poorly. JBF-LEG method provides good results. Therefore, the proposed fusion results obtained better quality and enhanced features in the fused image than other methods.

Finally, the fifth database of images is MR-T2, and SPECT obtained from the whole brain Harvard medical school, which is exhibited in Fig. 14 for the assessment of different fusion methods. The MRI image provides anatomical information, whereas the SPECT image provides a functional understanding of the human brain. To get both anatomical and functional data into a single resultant image, the source images are to be fused. Compared to other existing methods, the tumor regions are clearly enhanced by using the proposed method and obtaining good complementary and redundant information from source images. Furthermore, the proposed results reveal better fusion performance in terms of contrast, luminance, and clarity.

### Quantitative assessment

**Figure 15:**
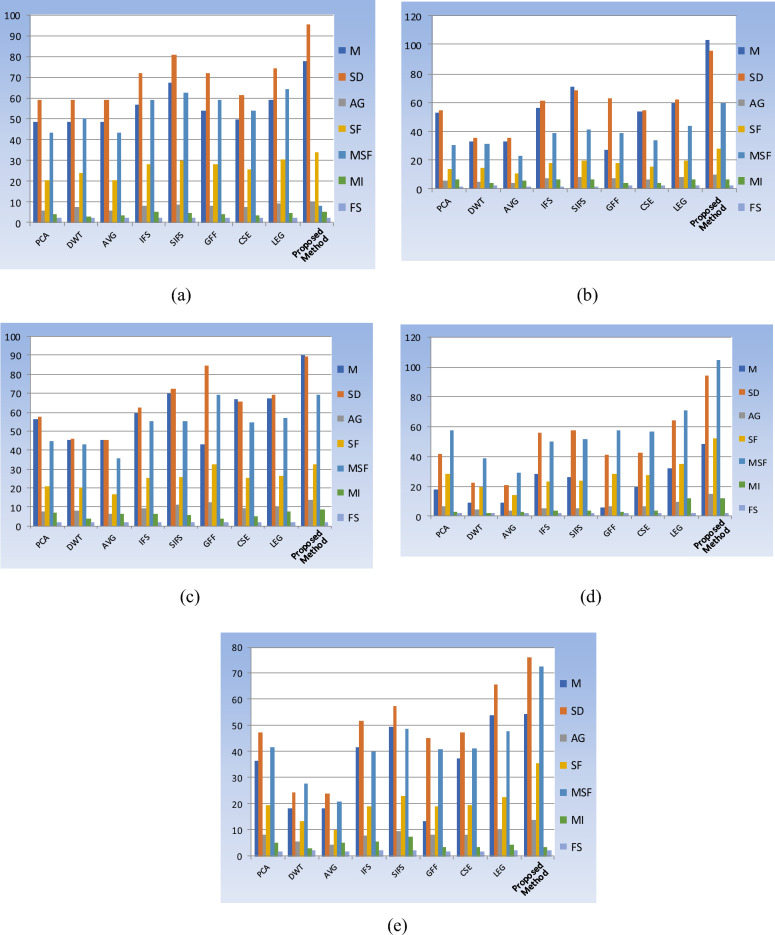
Graphical representation of fusion methods with Pair-1 in all databases: (**a**) database-1, (**b**) database-2, (**c**) database-3, (**d**) database-4, (**e**) database-5.

Visual evaluation alone cannot determine the quality of the integrated image. As a result, it is essential to calculate the fused image's objective values. The objective evaluation of eight existing fusion approaches and the proposed method on 16 pairs of medical datasets is shown in Tables 3, 4, 5, 6, 7. In each table, the highest value of each quality parameter is outlined in bold. The proposed fusion method provides better results and superiority over other methods. However, some of the quality metrics have low values in various databases, which are listed as follows: In database-1, MI value is in pair-3 (see Table 3). In database-2, MI value is in pair-2 (see Table 4). In database-4, MI and FS values are in pair-4 (see Table 6). In database-5, MI and FS values are in pairs 1 and 3 (see Table 7), MI value is in Pair-2 (see Table 6). Although the other pairs of databases have high values in the proposed method, they are observed in Tables 3, 4, 5, 6, 7. In this paper, the graphical representation of fusion methods with quality metric values for pair-1 in each database is shown in Fig. 15.

**Table 3:** Fusion results of various methods with performance metrics values for Database-1.

Database	Pair	Fusion methods	M	SD	AG	SF	MSF	MI	FS	COT
MR-T1 and MR-T2	1	PCA	48.5333	58.7312	5.7924	20.1823	43.0135	3.837	1.975	0.7004
		DWT	48.5525	59.0274	7.2338	23.9222	50.2048	2.8309	1.9732	1.0203
		AVG	48.5525	58.7064	5.801	20.2819	43.2194	3.2795	1.974	1120.1306
		IFS	56.6150	71.7527	8.2122	27.8113	58.8646	4.8538	1.9537	09,624
		SIFS	67.1062	80.5079	8.5481	29.4196	62.3853	4.2121	1.9597	12.2012
		GFF	53.5389	71.9251	7.864	28.1212	59.1134	3.7374	1.9813	0.3383
		CSE	49.517	61.3223	7.4495	25.4648	53.8682	3.3939	1.9706	0.4053
		LEG	59.0123	74.5067	9.0156	30.281	64.099	4.2573	1.9744	1.5663
		Proposed method	**77.6913**	**95.1587**	**9.4976**	**33.7491**	**70.7699**	**4.8547**	**1.9876**	1.5748
	2	PCA	40.9438	55.2071	4.2859	14.2257	30.5831	3.5405	1.9718	0.7118
		DWT	40.7866	55.1689	5.1176	16.2986	34.5273	2.2491	1.9723	0.9935
		AVG	40.7866	55.0178	4.2522	14.015	30.138	2.8657	1.9801	1394.2302
		IFS	45.3979	62.3180	5.9029	18.9957	40.6547	4.292	1.9259	0.7735
		SIFS	54.3761	74.3580	6.4803	21.5478	46.1618	3.6564	1.771	13.2598
		GFF	43.4384	72.565	5.6329	20.0051	42.836	2.8391	1.9888	0.1651
		CSE	43.209	59.6116	5.6557	18.8801	40.4665	3.244	1.9824	0.4279
		LEG	48.2613	66.2913	6.4226	20.6794	44.2119	3.4524	1.9664	1.5882
		Proposed method	**65.6529**	**89.4576**	**7.3426**	**25.3123**	**53.9790**	**4.4733**	**1.9911**	0.5793
	3	PCA	49.7700	59.2485	8.3181	24.0381	50.8087	4.0495	1.9798	0.6915
		DWT	48.8306	58.303	10.1202	27.5988	57.5019	**7.5856**	1.9788	1.1499
		AVG	48.8306	57.8606	8.1547	23.1832	49.0464	3.3622	1.9854	1559.7688
		IFS	62.2579	75.5283	11.7296	32.0522	69.5862	5.2866	1.9641	0.7214
		SIFS	71.5070	80.4278	12.1098	32.9983	71.5054	4.2527	1.928	11.2367
		GFF	50.9989	92.372	12.7505	37.3873	78.6519	2.9448	1.9931	0.2207
		CSE	53.9746	67.0591	10.9166	30.7088	64.683	3.7743	1.9785	0.3932
		LEG	62.1675	76.2289	12.2767	34.1826	71.9254	4.4006	1.9591	1.6309
		Proposed method	**84.1516**	**98.2910**	**13.2906**	**40.0876**	**83.7316**	5.0038	**1.9951**	0.5743
	4	PCA	35.495	46.1943	7.3978	3.4057	48.9533	3.2935	1.9551	0.7089
		DWT	35.7292	46.9029	9.7406	28.8464	59.5322	2.769	1.9756	1.1213
		AVG	35.7292	46.158	7.3558	23.3553	48.8729	3.2455	1.9505	1728.749
		IFS	54.2263	70.8374	10.6546	33.2495	69.3330	4.2621	1.8322	0.9278
		SIFS	63.1075	76.4565	10.8126	34.0487	70.9640	4.2265	1.8483	45.4125
		GFF	37.4758	75.0690	12.0071	36.0990	74.5683	3.0626	1.9660	0.1625
		CSE	35.6573	53.7194	10.1623	30.1351	63.089	2.9499	1.9946	0.4259
		LEG	54.0338	71.9469	12.0285	35.5139	73.9656	3.606	1.9780	1.6012
		Proposed method	**70.8957**	**92.5014**	**15.1002**	**47.5008**	**96.3242**	**5.1360**	**1.9949**	0.6033

Significant values are in bold.

**Table 4:** Fusion results of various methods with performance metrics values for Database-2.

Database	pair	Fusion methods	M	SD	AG	SF	MSF	MI	FS	COT
MRI-CT	1	PCA	52.7361	54.0957	5.3965	13.6861	30.3639	6.3918	1.5796	0.6783
		DWT	32.6739	35.3666	4.8506	14.243	31.0967	3.7058	1.6470	1.9144
		AVG	32.6739	34.9506	3.898	10.2469	22.6229	5.3013	1.6324	447.5107
		IFS	56.2292	60.8907	6.9227	17.6850	38.9477	6.3985	1.6103	1.0385
		SIFS	70.5806	68.7705	7.9889	19.3217	41.4694	6.3305	1.6156	41.8321
		GFF	27.3498	62.7113	7.0335	17.5453	38.6291	3.8193	1.6502	0.2001
		CSE	53.4047	54.3205	6.1695	15.332	33.817	3.475	1.651	0.4127
		LEG	59.6046	61.7562	7.6850	19.7906	43.3339	6.2153	1.651	1.5563
		Proposed method	**103.3001**	**95.8234**	**9.8195**	**27.5047**	**59.5506**	**6.5514**	**1.6511**	0.5841
	2	PCA	49.5394	61.4082	6.7699	17.1602	7.0703	4.2013	1.8245	0.8272
		DWT	39.4672	47.5308	6.8347	17.0778	36.1992	3.3937	1.9287	1.2686
		AVG	39.4672	47.2096	5.4013	13.0686	28.1275	3.851	1.9257	767.5424
		IFS	60.3590	73.8756	8.6043	21.5505	46.3240	5.2971	1.7877	0.8914
		SIFS	67.9338	75.4478	8.9402	22.1863	47.5899	**6.1245**	1.7891	50.2147
		GFF	39.026	86.9717	10.1792	24.9838	53.603	3.3865	1.9096	0.2117
		CSE	57.346	59.2109	7.9961	20.0395	43.1403	3.7216	1.8278	0.4368
		LEG	59.5622	74.1396	8.7713	22.0832	47.6133	5.7732	1.7632	1.6221
		Proposed method	**72.0662**	**90.8102**	**11.4689**	**30.8993**	**65.0788**	5.1573	**1.9653**	0.5972

Significant values are in bold.

**Table 5:** Fusion results of various methods with performance metrics values for Database-3.

Database	Pair	Fusion methods	M	SD	AG	SF	MSF	MI	FS	COT
T1 Weighted MR-MRA	1	PCA	56.5364	57.7863	7.6807	20.8249	44.7746	7.0121	1.9580	0.7795
		DWT	45.5719	45.8889	7.8457	20.3158	43.0089	3.9122	1.9218	1.203
		AVG	45.5719	45.487	6.4314	16.4252	35.3492	6.0534	1.9857	955.9763
		IFS	59.3350	62.4487	9.3827	25.3562	55.2508	5.9854	1.8319	1.1029
		SIFS	69.9219	72.0140	11.0313	25.9354	55.3563	5.2456	1.815	23.2478
		GFF	43.0042	84.4180	12.1798	32.2367	69.3424	3.9018	1.9437	0.1728
		CSE	66.6468	65.4174	9.2887	25.4313	54.5312	4.7298	1.7742	0.4156
		LEG	67.1919	69.4395	9.8698	26.4448	56.6842	7.4991	1.8445	1.5667
		Proposed method	**90.2692**	**89.5789**	**13.2688**	**32.6902**	**68.9848**	**8.6368**	**1.9934**	0.65

Significant values are in bold.

**Table 6:** Fusion results of various methods with performance metrics values for Database-4.

Database	Pair	Fusion methods	M	SD	AG	SF	MSF	MI	FS	COT
MRI-PET	1	PCA	17.8597	41.8299	6.8739	28.2244	57.7891	3.0028	1.8301	0.7104
		DWT	9.0069	21.9959	4.5906	19.4167	38.8851	2.056	1.9619	1.5526
		AVG	9.0069	21.0428	3.4507	14.1624	28.9981	3.0452	1.8469	2077.3853
		IFS	27.9597	55.7069	5.2418	23.2296	49.6730	3.5067	1.8373	1.22488
		SIFS	25.8475	57.5958	5.3715	23.9290	51.4897	3.2504	1.8703	43.0596
		GFF	5.4486	40.9076	6.8395	28.1165	57.5309	2.7795	1.8650	0.1891
		CSE	18.9799	42.2157	6.8364	27.7381	56.7449	3.7884	1.9498	0.858
		LEG	31.7304	64.3451	9.3575	34.6291	71.1669	11.725	1.9950	1.609
		Proposed method	**48.2289**	**93.8402**	**14.5608**	**52.5326**	**104.551**	**12.081**	**1.9955**	0.595
	2	PCA	25.8146	44.9239	4.7857	16.1999	35.2835	2.9769	1.7460	0.7054
		DWT	13.0062	22.7941	2.8792	9.8669	21.3932	4.1041	1.9806	1.8901
		AVG	13.0062	22..6096	2.4063	8.1422	17.7344	3.0243	1.7615	3101.4512
		IFS	37.4512	68.8663	5.8279	22.0309	47.4877	3.0095	1.8856	0.9637
		SIFS	49.4128	82.2304	6.8394	25.7063	55.9964	3.1047	1.9068	37.5467
		GFF	8.4668	42.972	4.5395	14.9047	32.5497	2.4121	1.7195	0.1892
		CSE	26.6921	45.5173	4.8645	16.3569	35.5782	1.9758	1.7779	0.8708
		LEG	43.6647	75.2944	6.4011	21.4454	46.9481	8.0772	1.9546	1.5864
		Proposed method	**64.0989**	**102.0984**	**14.7041**	**42.6956**	**90.6496**	**8.6682**	**1.9704**	1.6958
	3	PCA	32.2408	60.572	7.8963	26.3174	56.7006	2.8805	1.7074	0.7058
		DWT	16.2084	30.9837	4.9702	17.3932	37.0056	1.6998	1.7768	1.24
		AVG	16.2084	30.4342	3.9617	13.2006	28.4412	2.7635	1.7367	3370.0021
		IFS	32.2093	64.0469	7.3661	26.4777	56.8240	2.6624	1.8304	0.7631
		SIFS	47.5435	83.1326	8.4975	29.5320	63.7191	2.7858	1.8514	46.9472
		GFF	11.1531	57.5717	7.6434	25.2524	54.4338	2.4946	1.6968	0.2054
		CSE	32.5483	60.7098	77,977	26.0291	56.0739	2.2553	1.7183	0.8901
		LEG	43.641	77.3666	9.0136	27.1965	58.9266	2.861	1.7300	1.6755
		Proposed method	**52.4221**	**94.0823**	**13.5316**	**42.5302**	**89.4002**	**3.8450**	**1.9540**	1.0529
	4	PCA	62.8197	75.9753	14.5515	37.9157	80.7645	4.3382	1.6882	0.7122
		DWT	31.5647	38.778	9.1267	23.909	50.0066	**7.5787**	**1.9363**	2.457
		AVG	31.5647	38.1584	7.3002	19.0179	40.5119	4.5099	1.7435	3252.2631
		IFS	58.0666	78.3744	13.2005	37.4934	79.5276	4.8101	1.8464	0.7713
		SIFS	76.5504	95.2436	15.5735	41.9684	84.7232	5.0956	1.883	46.7710
		GFF	24.4786	70.6057	13.5961	34.9485	74.3632	3.1034	1.7007	0.1973
		CSE	62.942	75.9591	14.522	37.8286	80.5851	3.826	1.7025	0.8608
		LEG	85.4154	95.3379	16.7782	39.4183	84.4699	3.8656	1.6954	1.6021
		Proposed method	**79.9916**	**107.1076**	**25.2190**	**61.7708**	**126.451**	2.3511	1.7918	0.6474

Significant values are in bold.

**Table 7:** Fusion results of various methods with performance metrics values for Database-5.

Database	pair	Fusion methods	M	SD	AG	SF	MSF	MI	FS	COT
MRI-SPECT	1	PCA	36.2352	47.1111	8.1522	19.5749	41.6363	5.0262	1.7056	0.7826
		DWT	18.2216	24.0896	5.274	13.333	27.8491	2.6544	1.7735	1.6141
		AVG	18.2216	23.674	4.0893	9.8183	20.8843	5.0045	1.7132	2473.7608
		IFS	41.4692	51.6961	7.5899	18.8429	40.0965	5.4231	1.8931	0.4527
		SIFS	49.6941	57.3879	9.5927	22.9294	48.7513	**7.0542**	**1.9341**	102.9102
		GFF	13.228	45.0825	7.9466	19.1018	40.5689	3.3364	1.7378	0.1993
		CSE	37.2819	47.2817	8.0123	19.3176	41.054	3.1716	1.7553	0.8667
		LEG	53.7419	65.7482	9.6767	22.3273	47.5462	4.2158	1.7953	1.6746
		Proposed method	**54.0932**	**75.9647**	**13.8879**	**35.5462**	**72.4592**	3.4820	1.8550	0.6871
	2	PCA	34.8714	53.7192	6.47	19.0389	41.023	3.9957	1.8660	0.8005
		DWT	17.5435	24.2821	4.1407	12.7	26.989	2.5283	1.9368	1.5709
		AVG	17.5435	26.9825	3.2448	9.5456	20.5681	3.8844	1.8576	2340.863
		IFS	42.1777	59.3343	5.8476	18.2847	39.2935	4.7176	1.8309	0.4465
		SIFS	49.2064	64.6693	6.5153	20.2024	43.3789	5.027	1.8924	65.2178
		GFF	12.1432	51.3641	6.3202	18.544	39.937	3.026	1.8625	0.1812
		CSE	33.1598	47.7384	6.3418	18.4749	39.8046	2.0264	1.9630	0.8888
		LEG	55.802	75.469	8.0681	21.9276	47.3667	**8.4770**	1.9766	1.6518
		Proposed method	**73.3338**	**97.5903**	**11.7718**	**33.1091**	**69.7483**	7.4353	**1.9801**	0.5897
	3	PCA	35.1166	43.1009	6.9352	16.1679	34.1377	4.9323	1.8184	0.6914
		DWT	17.7122	21.9586	4.5147	10.8358	22.4341	4.6561	1.9316	1.1476
		AVG	17.7122	21.6992	3.4823	8.1164	17.1379	4.9244	1.8224	2582.2884
		IFS	48.0704	54.1787	6.6493	16.9438	35.8025	5.5147	1.8569	0.4573
		SIFS	57.2837	64.6756	7.5482	19.1225	40.3535	6.4363	1.8891	70.9837
		GFF	13.0797	41.0963	6.7428	15.7168	33.1096	3.4719	1.8179	0.1828
		CSE	41.4016	48.423	7.7312	17.9703	37.9366	2.1509	**1.9968**	0.875
		LEG	61.1308	66.9511	8.9721	19.545	41.3303	**6.5618**	1.9867	1.7004
		Proposed method	**61.1959**	**74.3541**	**12.771**	**29.3016**	**59.6331**	2.8542	1.9025	0.6292
	4	PCA	41.8842	58.4375	7.7928	21.5199	45.7955	4.934	1.7545	0.686
		DWT	21.0604	29.727	5.0281	14.3692	29.9761	3.1448	1.9263	1.4523
		AVG	21.004	29.3633	3.9093	10.7944	22.9718	5.414	1.7845	2717.7096
		IFS	40.2397	56.1887	6.5095	18.9695	40.2515	5.4551	1.8819	0.4298
		SIFS	51.2304	66.9755	8.0639	23.2374	49.5365	6.0178	1.8735	0.3455
		GFF	14.7627	54.9067	7.5395	20.7558	44.0986	3.1970	1.7971	0.2234
		CSE	38.9001	51.7263	7.4131	20.4281	43.4475	2.1406	1.9302	0.8763
		LEG	60.8555	79.3465	9.2174	24.5329	52.2363	4.4507	1.8134	1.6981
		Proposed method	**61.2958**	**86.9105**	**13.1782**	**36.5818**	**74.6347**	**7.2719**	**1.9315**	0.6266
	5	PCA	48.847	65.3127	7.411	24.9597	52.9366	3.2219	1.5295	0.6767
		DWT	24.4395	33.0312	4.8265	16.0127	33.4142	1.8329	1.5506	1.425
		AVG	24.4395	32.6755	3.7079	12.487	26.4838	6.6461	1.7782	2869.5802
		IFS	46.9477	62.3808	7.2338	24.0164	50.9765	3.8704	1.6414	0.4321
		SIFS	56.2979	71.0858	8.4262	29.3485	62.1308	4.394	1.6217	0.3467
		GFF	20.5437	60.547	7.409	23.7231	50.3315	2.6472	1.5469	0.1987
		CSE	48.2853	61.1433	7.5909	24.7274	52.8658	1.2047	1.6022	0.8681
		LEG	54.4278	69.9056	8.1900	24.302	51.9539	7.1156	1.8553	1.6624
		Proposed method	**74.7042**	**92.1095**	**12.785**	**38.6877**	**79.9567**	**8.6589**	**1.8750**	0.5906

Significant values are in bold.

The proposed fusion method obtained remarkable performance, especially database-1 of pair-1 and pair-4, database-2 of pair-1, database-3 of pair-1, database-4 of pair-1, and database-5 of pair-5, and extracted the required details from the source images to produce a fused image with good contrast. Ultimately, the proposed fusion method removes the vagueness and uncertainties in the fused image and gives better-quality results than the other fusion methods.

## Conclusion

This article presents a novel approach for medical image fusion using a pythagorean fuzzy set for better clinical diagnosis. The core concept of our proposed method is mainly described as four stages. In the first stage, the source images were decomposed into base and detail layer images using two-scale decomposition. Then, the base layers of two source images were fused using a pythagorean fuzzy set for the extraction of more structural information. After that, the SF was employed to fuse the detailed layers. Finally, the base and detailed fused images were combined to obtain an enhanced fused image. In this paper, we used five medical databases for the fusion process, and experimental results are proving its superiority both visually and quantitatively compared to other methods.

An experimental result proves the quality of the proposed method in terms of visual and objective assessments, as shown in Figs. 10, 11, 12, 13, 14 and Tables 3, 4, 5, 6, 7. In Fig. 10, Pair-4 of database-1 shows that the edges and overall clarity of the proposed fused image are good compared to other methods, and quantitatively high values are 15.1002 for AG and 47.5008 for SF from Table 3. Moreover, the tumor region is clearly enhanced in Fig. 13, and has a quantitatively high value in pair-1 (93.8402 for SD, 14.5608 for AG, 52.5326 for SF, and 1.9955 for FS) from Table 6. As aforementioned, the positive aspects of the proposed method provide a good contrast fused image without artifacts compared to other methods and removes uncertainties. This method is suitable for grayscale and color medical images. In the future, the extension of work will be based on novel fuzzy sets and fusion rules for better diagnosis.

## Data Availability

The datasets analyzed during this work are made publicly available, and was obtained from whole brain atlas website https://www.med.harvard.edu/aanlib, and google drive https://drive.google.com/drive/folders/0BzXT0LnoyRqleHhrdzE3UUVzdVE?resourcekey=0-kPMk3Pjq10up3Rrxjp5Rwg.
